# PPARγ and TGFβ—Major Regulators of Metabolism, Inflammation, and Fibrosis in the Lungs and Kidneys

**DOI:** 10.3390/ijms221910431

**Published:** 2021-09-28

**Authors:** Gábor Kökény, Laurent Calvier, Georg Hansmann

**Affiliations:** 1 Institute of Translational Medicine, Semmelweis University, 1089 Budapest, Hungary; 2 International Nephrology Research and Training Center, Semmelweis University, 1089 Budapest, Hungary; 3 Department of Molecular Genetics, University of Texas (UT) Southwestern Medical Center, Dallas, TX 75390, USA; calvier.laurent@gmail.com; 4 Center for Translational Neurodegeneration Research, University of Texas (UT) Southwestern Medical Center, Dallas, TX 75390, USA; 5 Pulmonary Vascular Research Center (PVRC), Hannover Medical School, 30625 Hannover, Germany; 6 Department of Pediatric Cardiology and Critical Care, Hannover Medical School, 30625 Hannover, Germany

**Keywords:** PPARγ, pulmonary arterial hypertension, TGFβ, vascular injury, inflammation, proliferation, kidney fibrosis

## Abstract

Peroxisome proliferator-activated receptor gamma (PPARγ) is a type II nuclear receptor, initially recognized in adipose tissue for its role in fatty acid storage and glucose metabolism. It promotes lipid uptake and adipogenesis by increasing insulin sensitivity and adiponectin release. Later, PPARγ was implicated in cardiac development and in critical conditions such as pulmonary arterial hypertension (PAH) and kidney failure. Recently, a cluster of different papers linked PPARγ signaling with another superfamily, the transforming growth factor beta (TGFβ), and its receptors, all of which play a major role in PAH and kidney failure. TGFβ is a multifunctional cytokine that drives inflammation, fibrosis, and cell differentiation while PPARγ activation reverses these adverse events in many models. Such opposite biological effects emphasize the delicate balance and complex crosstalk between PPARγ and TGFβ. Based on solid experimental and clinical evidence, the present review summarizes connections and their implications for PAH and kidney failure, highlighting the similarities and differences between lung and kidney mechanisms as well as discussing the therapeutic potential of PPARγ agonist pioglitazone.

## 1. Introduction

Peroxisome proliferator-activated receptors (PPARs; α, β/δ, γ) are ligand-activated transcription factors of the nuclear receptor superfamily that regulate metabolic homeostasis of the cell. Among them, PPARγ regulates synthetic metabolism (anabolism) in the adipose tissue and plays an important role in glucose metabolism [1] and cardiac development [2]. The human PPARγ gene contains nine exons spanning over 100 kilobases located on chromosome 3 [3]. The ligand-activated PPARγ regulates target genes by forming a heterodimer with the retinoid X receptor (RXR). Mutations in PPARγ gene have been associated with dysfunctional lipid and glucose homeostasis leading to obesity and type 2 diabetes mellitus (T2DM) [4,5] but also with thyroid cancer [6].

Although PPARγ is predominantly a key regulator of adipocyte homeostasis, it is ubiquitously expressed. Overall, there were predominantly protective effects in the cardiovascular system, including systemic and pulmonary circulation. The diseases and conditions which are positively affected by PPARγ activation in preclinical and/or clinical studies include but are not limited to pulmonary arterial hypertension (PAH), prediabetes/insulin resistance, cardiovascular diseases such as stroke in prediabetes, nephrotic syndrome, kidney, or lung fibrosis, independently of the blood glucose lowering effect [7,8,9,10,11,12].

Recently, post-transcriptional regulation of PPARγ by microRNAs have been implicated in different diseases [10,13,14]. Protein phosphorylation is another regulatory mechanism that can reduce or increase the transcriptional activity of PPARγ [15].

Since 2007 [16], PPARγ agonists have emerged as promising novel, antiproliferative, anti-inflammatory, insulin-sensitizing, and efficient medications for the treatment of PAH. Still, the results of earlier diabetes studies and their false interpretations, as well as scarce reports on the possible adverse effects, substantially diminished the interest on using pharmacological PPARγ activation for the treatment of cardiovascular diseases, including PAH. However, the recent, very large IRIS trial [17,18,19] did not confirm any serious adverse effects for the PPARγ agonists pioglitazone when used in patients with insulin resistance/prediabetes—in fact, pioglitazone decreased the risk for stroke and myocardial infarction [17]. The present review summarizes recent experimental and clinical evidences showing how PPARγ participates in the pathogenesis of pulmonary and renal diseases while also highlighting the therapeutic potential of the thiazolidinedione (TZD) class PPARγ agonists (e.g., pioglitazone and rosiglitazone) in these diseases.

## 2. Role of PPARγ Crosstalk with TGFβ Superfamily Members and microRNAs in Pulmonary Vascular Homeostasis

The pathology of PAH affects not only the pulmonary arteries but also several extrapulmonary organs (heart, skeletal muscle, and adipose tissue) [20,21,22,23,24] that share common metabolic abnormalities (i.e., suppression of mitochondrial glucose oxidation and increased glycolysis, disturbed fatty acid oxidation (FAO), and dyslipidemia/insulin resistance) [16,20,24,25,26].

PPARγ regulates several target genes that are strongly implicated in the pathobiology of PAH, for instance adiponectin (APN), IL-6, monocyte chemotactic protein-1 (MCP-1/CCL2) or endothelin-1 (ET-1) [25,27]. PPARγ agonists have been proven to exert antiproliferative (on vascular smooth-muscle cells (VSMC)), anti-inflammatory, proangiogenic, and proapoptotic effects in cells, animal models, and patients, emphasizing their therapeutic potential in PAH and other cardiopulmonary diseases, even in the absence of insulin resistance [25].

Bone morphogenetic protein 2 (BMP2) is a ligand of BMPR2 and inhibits VSMC growth. In endothelial cells, however, BMP2 acts as a survival factor and hence may counteract the endothelial cell injury and dysfunction in the early stages of PAH. Loss-of-function mutations in the BMPR2 gene are frequently seen in familial/hereditable (HPAH, 70%, i.e., germline mutations) and idiopathic PAH (IPAH, 10–20%) cases. The recent discovery of an antiproliferative BMP2/BMPR2-PPARγ-ApoE axis [28] in VSMC suggests that dysfunction of BMPR2 reduces endogenous PPARγ activity [28]. Thus, the activation of PPARγ might reverse the PAH phenotype in patients with or without BMPR2 mutations. Pulmonary BMPR2 expression decreases even in the absence of BMPR2 mutations in idiopathic or HPAH and in PAH secondary to connective tissue disease or congenital heart disease [29]. Importantly, PAH patients have reduced pulmonary BMP2 [30], PPARγ [31], and apolipoprotein E (ApoE) mRNA expression [30]. PPARγ inhibits cell growth in hypoxia-exposed human pulmonary arterial smooth-muscle cells (HPASMC) through the suppression of miR-21, and its activation cancels programmed cell death protein 4 (PDCD4) repression, thus facilitating the apoptosis of HPASMC [32]. SCUBE1, a proposed BMP co-receptor has been recently identified as a novel factor in the pathogenesis of PAH. In cultured PAECs, BMPR2 knockdown induced SCUBE1 downregulation, and both plasma and lung biopsy samples of PAH patients demonstrated reduced SCUBE1 expression that correlated with disease severity [33].

The calcineurin inhibitor tacrolimus (FK506) used in picomolar concentrations binds to the BMP signaling repressor FK-binding protein-12 (FKBP12). Low-dose FK506 treatment of floxed endothelial cell-specific Bmpr2^−/−^ mice prevented the development of hypoxia-induced pulmonary arterial muscularization and normalized RVSP. Additionally, a 3 week FK506 treatment was able to reverse established PAH in the SU5416 (VEGFR2 inhibitor)/hypoxia (SuHx) rat model via the activation of apelin that suppresses PASMC proliferation. In human PAECs obtained from iPAH patients, low-dose FK506 reduced endothelial dysfunction [34].

**Figure 1:**
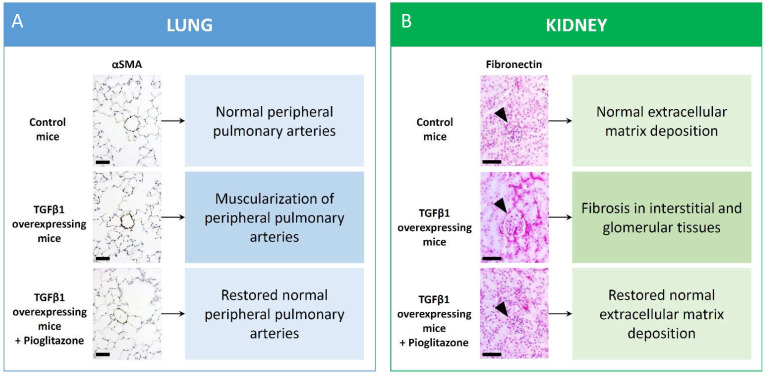
Representative photomicrographs of lung and kidney immunohistochemistry in TGFβ overexpressing mice treated with pioglitazone. Lungs stained for αSMA depicted significant muscularization of peripheral pulmonary arteries in untreated TGFβ overexpressing mice as compared to controls but restored arterial wall morphology upon pioglitazone treatment (**A**). Scale bar: 50 µm. Fibronectin staining of the kidneys in untreated TGFβ overexpressing mice depicts increased tubulointerstitial and glomerular production (arrowhead points on glomeruli) but restored fibronectin content after chronic pioglitazone treatment (**B**). Scale bar: 50 µm.

We identified PPARγ as a missing link and a key regulator of the functional antagonism between BMP2 and TGFβ1 pathways in human and murine VSMC [10,14]. In HPASMC, PPARγ activation with pioglitazone inhibited a novel noncanonical TGFβ1-pSTAT3-pFoxO1 pathway, in addition to the inhibition of the canonical TGFβ1-pSmad3/4 axis [10,35]. Additionally, pioglitazone treatment of TGFβ1-overexpressing mice reversed PAH and pulmonary vascular remodeling [10] (Figure 1). Recently, the alleviation of disrupted PPARγ-p53 axis in PAEC from BMPR2 mutant patients emerged as a possible therapeutic potential for PAH [36]. Even in the absence of other possible injuries the cell-specific deficiency of PPARγ in VSMCs was demonstrated to increase pulmonary vascular muscularization in mice, independently of a low-fat or high-fat diet [37].

It has been shown that the miR-130/-301 family promotes pulmonary hypertension through systemic regulation of miRNA networks [38,39,40], where PPARγ plays a key role as a direct target of this miRNA family. For instance, pulmonary arteries from IPAH patients demonstrated increased miR-130a/-301b expression as compared to controls [10]. Additionally, TGFβ1 stimulation of HPASMC reduces PPARγ-mRNA via miR-130a/-301b, hence suppressing the BMP2/BMPR2-PPARγ axis. Recently, new miRNAs upregulated by the BMP2/PPARγ axis have been identified. In HPASMC, BMP2 induces miR-331-5p, which downregulates the mRNA expression of the platelet isoform of phosphofructokinase (PFKP), a rate-limiting enzyme of glycolysis and pro-proliferative factor that is highly expressed in situ in pulmonary arteries of IPAH patients vs. controls [10]. Activation of the BMP2/BMPR2-PPARγ axis upregulates miR-331-5p and miR-148a (suspected to repress cell proliferation), thus inhibiting proliferation and glucose metabolism in VSMC [10,14].

Heat-shock protein 90 (Hsp90) is a molecular chaperone involved in many cellular protein interactions, and abnormal Hsp90 expression has been recently attributed to PAH [41,42]. Increased expression levels of cytosolic Hsp90 have been found in PASMCs of PAH patients, and a Hsp90-inhibitor suppressed PASMC proliferation [42]. Targeted inhibition of mitochondrial Hsp90 reversed pulmonary arterial remodeling in the monocrotaline rat model of PAH and in PAH-PASMC in vitro [41]. Hsp90 might also have a strong cellular interplay with PPARγ. Interestingly, Hsp90 stabilized PPARγ in both liver cells [43] and adipocytes [44], and Hsp90 inhibition lowered PPARγ levels, while Hsp90 overexpression diminished PPARγ degradation [43] in liver cells. However, the reduced Hsp90/eNOS signaling and endothelial dysfunction in PAH has been attributed to reduced PPARγ levels, modulated by miR-27b overexpression in HPAECs and also in monocrotaline-induced rat model of PAH [45]. The exposure of ovine PAECs to TGFβ1 resulted in reduced PPARγ expression, mitochondrial dysfunction, and disrupted Hsp90/eNOS signaling [46]. These studies suggest that dysfunctional, boosted TGFβ1 results in suppression of the PPARγ/Hsp90/eNOS signaling, contributing to endothelial dysfunction and PASMC proliferation in PAH.

**Figure 2:**
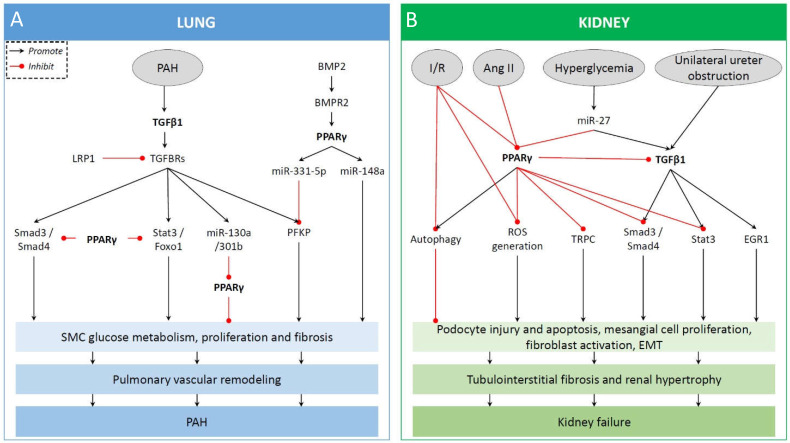
Summary of PPARγ actions in pulmonary arterial hypertension (**A**) and kidney disease models (**B**).

LRP1 is a recognized vasoprotective receptor that interacts with several ligands, such as growth factors, cytokines, lipoproteins, and extracellular matrix components. LRP1 serves as a co-receptor for TGFBRs inhibiting the growth effect of TGFβ by interacting with Smad2/3 signaling [47]. Reduced vascular LRP1 expression was recently demonstrated in human PAH, and LRP1 in VSMC was found to protect from PAH in vivo [48]. Importantly, the activation of PPARγ by pioglitazone reversed PAH caused by LRP1 deficiency in murine VSMC, inhibiting Smad3, Nox4, and CTGF [48]. Hence, PPARγ activation can normalize TGFβ1/BMP2 homeostasis via regulation of both canonical and non-canonical TGFβ1 pathways and the expression of key miRNAs involved in cell proliferation and glucose/lipid metabolism (summarized in Figure 2).

## 3. Dysregulation of Metabolic Pathways and PPARγ Dysfunction in PAH

The role of dysfunctional PPARγ in the pathogenesis of metabolic disturbances has been demonstrated both in human PAH and in experimental models. In patients suffering from idiopathic pulmonary arterial hypertension, PPARγ mRNA expression was found to be markedly reduced in the failing RV [49]. Knockdown of PPARγ in cultured HPASMC has been associated with reduced PGC1α and with stimulating mitochondrial fragmentation and superoxide production and inducing proliferation [50].

The elevated TG/HDL ratio in PAH patients is the manifestation of lipid and lipoprotein homeostasis alterations due to insulin resistance [20,24]. Decreased fatty acidy oxidation (FAO) can directly cause myocardial lipid accumulation (lipotoxicity) [51], and this occurs in end-stage human PAH-RVs [42] as well as in the SU5416 (VEGFR2 inhibitor)/hypoxia (SuHx) PAH rat model [49]. In addition, the targeted deletion of PPARγ in cardiomyocytes of mice induces biventricular systolic dysfunction even in the absence of PAH [49]. Oral treatment in the SuHx rat model with PPARγ agonist pioglitazone reverses PAH and prevents RV failure through regulating mRNA and miRNA networks that restore mitochondrial fatty acid oxidation (FAO) and prevent lipotoxicity [49]. Studies in cardiomyocytes identified a direct link between miR-197 and miR-146b overexpression and the suppression of genes that drive FAO. PPARγ activation downregulated miR-197 and miR-146b that were upregulated in the SuHx-RV but were also found to be upregulated in the pressure-overloaded failing human RV in end-stage idiopathic PAH [49]. Thus, PPARγ activation could prevent lipotoxicity by normalizing transcriptional and post-transcriptional regulation of the disturbed lipid metabolism and mitochondrial function.

BOLA3 (BolA Family Member 3) is a member of mitochondrial iron-sulfur cluster assembly system. BOLA3 deficiency has been recently attributed to PAH via the activation of glycolysis and fatty acid oxidation, inhibiting glycine catabolism and increasing mitochondrial respiration in PAEC [52]. In cultured PAECs but not PASMCs, hypoxia downregulated BOLA3 expression. In addition, BOLA3 was found to be repressed in lungs of hypoxic C57Bl/6 mice and the SuHx rat PAH model, and in lung biopsies of PH patients. Importantly, orotracheal administration of adeno-associated virus carrying BOLA3 transgene was able to prevent hypoxia-induced PH in mice [52]. In human brown adipose tissue, BOLA3 gene expression was found to be positively correlated to PPARG expression [53].

Tribbles homolog 3 (TRIB3), a pseudokinase of the Tribbles family that inhibits AKT phosphorylation, is involved in several metabolic cellular events in the liver or adipose tissue [54,55]. TRIB3 also plays a role in the development of skeletal muscle insulin resistance and cellular glucotoxicity in diabetes [56]. Recently, TRIB3 was recognized to participate in the pathogenesis of pulmonary hypertension by reducing PPARγ activity [57]. In cultured PAECs, lentiviral overexpression of TRIB3 upregulated ERK1/2 and downregulated PPARγ and eNOS activity under both normoxia and hypoxia. Knockdown of TRIB3 by 50% in hypoxic PAECs reduced ERK1/2 and increased eNOS phosphorylation. The early pioglitazone treatment of rats with hypoxia-induced pulmonary hypertension (HPH) partially ameliorated PH and vascular insulin resistance through reduction of TRIB3 and ERK1/2 activity; pioglitazone also restored eNOS [57]. These findings suggest that hypoxia-induced TRIB3 and insulin resistance in PAECs contributes to PH that can be inhibited by early activation of PPARγ.

## 4. PPARγ in Renal Glomerular and Epithelial Cell Metabolism

PPARγ protein is expressed in several regions of the kidney, including different renal tubule segments [58], interstitial cells, the juxtaglomerular apparatus, podocytes, mesangial cells, and renal microvascular endothelial cells [59]. Since multiple renal cells show endogenous PPARγ expression and activity, PPARγ might play an important role in maintaining normal homeostasis and function of the kidney. Several studies on synthetic PPARγ agonists showed renoprotective effects of such compounds in both diabetic and nondiabetic kidney diseases and models of renal fibrosis [11,60,61,62]. PPARγ agonists of the thiazolidinedione class (“TZDs”), such as pioglitazone and rosiglitazone, have been demonstrated to induce PPARγ mRNA and protein expression in podocytes and tubular epithelial cells, in association with the amelioration of aging-related progressive renal injury [63,64]. The effects of PPARγ activation on experimental kidney disease models are summarized in Figure 2.

In the renal glomerulus, glucose and free fatty acids (FFA) are freely filtrated. Approximately 70% of filtrated FFAs are reabsorbed and then metabolized by β-oxidation within mitochondria in the proximal tubules, providing a significant energy source (in form of ATP); on the other hand, high amounts of intracellular fatty acids might limit ammonia production [65]. Mice deficient of PPARγ (having disrupted exon B1 of PPARγ2) and leptin develop metabolic syndrome with dyslipidemia, as well as renal hypertrophy and increased expression of the profibrotic TGFβ in the kidney [66]. Similar to FFA, filtrated glucose is also reabsorbed in the proximal tubules, using sodium-dependent glucose cotransporters (SGLT2 located in segment S1 and SGLT1 in segment S3). Hyperglycemia results in dysfunction of the SGLT-mediated glucose reabsorption in proximal tubular cells and promotes the profibrotic epithelial-to-mesenchymal transition (EMT). Such hyperglycemia-induced EMT can be reversed by PPARγ agonists that restore the SGLT-mediated glucose reabsorption [67].

In a recent study, PPARγ was shown to regulate proximal tubule cell metabolism by suppressing glycolysis and EGF degradation. Indeed, inhibition of PPARγ with GW9662 resulted in proximal tubule cell dysfunction in vitro, and in C57Bl6 mice it caused tubular hypertrophy, increased interstitial collagen deposition, and expression of kidney injury molecule-1 (KIM-1) [68]. These findings implicate that PPARγ agonists might exhibit their antifibrotic effect in the kidneys—at least partly—via modulation of tubular epithelial cell metabolism.

Podocytes play a principal role in the glomerular filtration and also express PPARγ [69]. Fatty acid treatment of podocytes (as a lipotoxicity model) tended to reduce PPARγ expression and led to inflammatory and apoptotic cellular events [70]. Several animal models of podocyte injury revealed the protective effect of PPARγ in podocytes. For instance, in puromycin aminoglycoside (PAN)-induced podocyte damage (that leads to nephrotic syndrome), pioglitazone treatment reduces proteinuria to the same extent as high dose glucocorticoid treatment and effectively attenuates podocyte damage [8]. The protective effect of PPARγ activation in podocytes is attributed to the reduction of profibrotic TGFβ expression and inhibition of apoptosis [64], restoring podocyte synaptopodin expression and ameliorating podocyte foot process effacement [62]. Rosiglitazone reduced aldosterone-induced podocyte damage by restoring nephrin expression and slit diaphragm integrity, as well as by reducing the amount of oxidative radicals [71]. Rosiglitazone also ameliorated the stretch-induced decrease in nephrin expression of podocytes in vitro [72]. Recently, fibroblast growth factor-1 (FGF1) has been demonstrated to reduce TGFβ expression via the induction of PPARγ, which resulted in EMT inhibition on cultured mouse podocytes and diabetic mouse model, reducing fibrosis and proteinuria [73].

One of the mechanisms of how PPARγ can reduce proteinuria and glomerular disease has been demonstrated lately by Sonneveld and colleagues. Transient receptor potential channel C6 (TRPC6) is a nonspecific calcium (Ca^2+^)—conducting ion channel and a transcriptional target of PPARγ and reduced TRPC6—mediated Ca^2+^ influx into podocytes leads to podocyte injury in glomerular disease. Cultured mouse podocytes were treated with pioglitazone or rosiglitazone, which inhibited PAN and adriamycin-induced TRPC6 overexpression and significantly inhibited TRPC6 promoter activity. In vivo, rats treated with pioglitazone developed less podocyte damage and milder albuminuria in an adriamycin-induced nephropathy model [74]. Thus, the activation of PPARγ can restore glomerular function by reducing podocyte damage. Apart from the damaged podocytes, glomerular mesangial cells also play pivotal role in the pathogenesis of glomerular sclerosis and function loss. PPARγ activation in cultured rat glomerular mesangial cells decreased AngII-induced Ca^2+^ influx via reducing TRPC activity, inhibiting mesangial cell proliferation, one of the hallmarks of glomerulosclerosis [75].

Of note, activation of pioglitazone as additional treatment over immunosuppression in a child with refractory nephrotic syndrome reduced proteinuria and increased eGFR, while less immunosuppression was needed to maintain renal function [8]. These studies emphasize the critical role of PPARγ in the regulation of renal epithelial, mesangial cell, and podocyte metabolism and homeostasis.

## 5. PPARγ in Kidney Fibrosis

Fibroproliferative diseases are estimated to account for up to 45% of mortality worldwide [76], resulting in high demand for new therapies fighting tissue fibrosis. PPARγ agonists emerged in the last decade as such new therapies: reduced albuminuria and nephropathy were observed in T2DM patients treated with TZD-class PPARγ agonists [77].

Epiblast-specific systemic deletion of the PPARγ gene in mice leads to the spontaneous development of T2DM and renal fibrosis in aging mice with glomerular hypertrophy, significant proteinuria and collagen deposition. Interestingly, this is associated with antiphospholipid syndrome, glomerular immune complex deposition, and macrophage infiltration [78]. On the other hand, hyperglycemia was shown to decrease PPARγ activity, associated with the upregulation of miR-27a [79]. MiR-27a represses PPARγ and activates TGFβ/Smad3 signaling leading to tubulointerstitial fibrosis, and both in diabetic rats and patients, the elevated plasma miR-27a was associated with poor renal function [80]. Inhibition of miR-27a both in cultured rat mesangial cells and in streptozotocin-induced diabetic rats (a T1DM model) abrogated the reduction of PPARγ and in vivo decreased renal ECM accumulation and podocyte injury [79]. Pioglitazone treatment of ZDF rats, a model of human T2DM, ameliorated diabetic kidney disease and reduced blood pressure as well as interstitial collagen-I and TGFβ production, which was associated with lower renal expression of Twist-1, an evolutionarily conserved protein that can accelerate renal epithelial-to-mesenchymal transition (EMT) and interstitial fibrosis [81].

Furthermore, several experimental studies show that PPARγ agonists bear antifibrotic effects independent of glycemic control. For instance, in the lung fibrosis model induced by silica exposure in mice, a PPARγ agonist inhibited both the reduction of pulmonary PPARγ and LXRa as well as the increase in TGFβ, fibronectin, and collagen-I expression [82]. Further, PPARγ agonist treatment prevented interstitial fibrosis and inflammation in unilateral ureter obstruction (UUO) mouse model of kidney fibrosis through reduction of renal TGFβ expression [9]. It was recently demonstrated that PPARγ activation in TGFβ transgenic mice inhibits the TGFβ-STAT3 and TGFβ-EGR1 transcriptional activation pathways, thus preventing renal fibrosis induced by elevated circulating TGFβ [11] (Figure 1). In kidney fibrosis, the elevated angiotensin-II levels also reduce renal PPARγ expression both in vivo and in vitro, while the angiotensin-II receptor blocker losartan exerts its renoprotective effects partly via the upregulation of PPARγ [83]. Repression of the TGFβ/Smad signaling by PPARγ agonist treatment was recently demonstrated in the hyperuricemia-induced rat model of renal fibrosis, associated with reduced proteinuria, serum creatinine, and BUN levels as well as interstitial ECM accumulation [84]. Another in vivo study where massive glomerular damage and renal fibrosis has been induced with subtotal nephrectomy in rats has implicated the beneficial effect of combined pioglitazone and angiotensin receptor blocker treatment over monotherapies in preserving podocytes, reducing glomerular macrophage infiltration and tubulointerstitial fibrosis. Intriguingly, pioglitazone—even in monotherapy—was able to reduce glomerulosclerosis [85].

Several in vivo and in vitro models emphasize the antifibrotic, TGFβ1-antagonizing effect of BMP7/ALK3 (activin-like kinase-3). For instance, administration of human recombinant BMP7 to rats subjected to UUO or mice with chronic glomerulonephritis reversed the fibrotic process and tubular damage via increased Smad1/5 signaling and reduced Smad2/3 phosphorylation, counteracting the canonical TGFβ1 signaling [86,87]. The induction of BMP signaling via ALK3 activation also inhibits renal fibrosis and tubular epithelial damage in mouse models of renal ischemia-reperfusion, UUO, or glomerulonephritis [88]. In a recent study, the administration of low-dose FK506 inhibited UUO-induced renal fibrosis in mice and activated ALK3 via ARNT transcription factor in cultured tubular epithelial cells, suggesting the antifibrotic role of FKBP12/ARNT/ALK3/BMP7 signaling [89]. Additionally, BMP7 increased both PPARγ expression and activity in cultured human mesangial cells, and the PPARγ agonist rosiglitazone reduced TNFα induced mesangial cell damage in vitro [90].

Fibroblast activation and proliferation is a key step in kidney fibrosis. PPARγ agonist treatment of primary mouse renal fibroblast suppressed PDGF-induced proliferation by inhibiting AKT phosphorylation and subsequent skp2 expression, which regulates cell proliferation via inhibition of p21/p27 effects blocking cell cycle progression [91]. Recently, it has been demonstrated that PPARγ-HGF production in renal fibroblasts regulates tubular epithelial cell survival. Pioglitazone treatment of cultured fibroblasts induced HGF expression, and conditioned media of these fibroblasts significantly attenuated staurosporine-induced acute epithelial cell injury and apoptosis in vitro, but this effect was abrogated by inhibition of downstream HGF signaling [92].

PPARγ activity has been attributed to a healthy epithelial phenotype of proximal tubular epithelial cells, inhibiting EMT and fibrogenesis. The induction of EMT and interstitial collagen production due to unilateral ureter obstruction (UUO) in mice could be attenuated by PPARγ agonist rosiglitazone, which preserved the proximal tubular cell phenotype [93]. In a recent study, the beneficial effect of PPARγ activation was attributed to increased renal Klotho expression and reduced oxidative stress, which effectively ameliorated the age-related nephrosclerosis in ApoE-null mice [94]. Interestingly, mice with Klotho gene loss of function mutations (kl/kl mice) develop cardiac hypertrophy associated with increased cardiac TGFβ protein expression [95].

## 6. PPARγ in Renal Inflammation and Cardiovascular Disease

In hyperoxaluric mouse model, pioglitazone suppressed renal calcium-oxalate (CaOx) crystal formation and inflammatory injury by enhancing the PPAR-γ mediated expression of miR-23, which dampened macrophage polarization to inflammatory (M1) phenotype but induced the anti-inflammatory M2 phenotype [96]. In a different model, distal tubules of rats that were treated with ethylene glycol to induce CaOx formation, rosiglitazone reduced CaOx crystal formation, oxidative stress, and TGFβ signaling. Similar results were obtained in vitro, using canine distal tubule cells that were induced with oxalate [97].

Interestingly, mice having a macrophage-specific deletion of PPARγ or RXRa develop lupus-like autoimmune glomerulonephritis and antinuclear antibodies [98]. The anti-inflammatory effect of PPARγ raises the therapeutic potential of PPARγ agonists such as pioglitazone in the prevention of chronic rejection after kidney transplantation (see below). The possible role of PPARγ in the development, severity, or progression of glomerulonephritis has been confirmed by another study using a different approach: When podocyte-specific PPARγ-deficient mice were challenged with anti-GBM nephrotoxic serum, they developed more severe glomerulonephritis with mononuclear cell infiltration as compared to wild-type mice treated with same nephrotoxin. Additionally, human kidney biopsies from patients with rapid progressing glomerulonephritis (RPGN) depicted the absence of PPARγ in the nuclei of cells in affected glomeruli [99].

Cardiovascular disease due to arterial calcification is a major complication in chronic kidney disease patients. One of the leading pathomechanism is hyperphosphatemia-induced arterial calcification and differentiation of VSMC into osteoblasts [100]. Hyperphosphatemia reduced PPARγ and Klotho expression in bovine aortic VSMCs, which were reversed by rosiglitazone treatment [101]. Decreased PPARγ expression was recently associated with hyperphosphatemia-induced osteogenic VSMC differentiation in CKD patients, too, and also in mouse VSMC cell line, where reduced BMP2 expression accompanied reduced PPARγ. Here, rosiglitazone inhibited calcification in vitro and also inhibited the hyperphosphatemia-induced vascular calcification in a mouse model of CKD, and this effect was Klotho dependent [102]. Thus, the PPARγ-Klotho axis plays an important role in the hyperphosphatemia-induced ossification of arterial VSMCs. In addition, recent experimental data suggest that PPARγ also plays a protective vascular role against atherosclerosis development by maintaining vascular homeostasis and reducing vascular inflammation. The long-term pioglitazone treatment of ApoE-null mice (a known model for advanced atherosclerosis) markedly reduced the total atherosclerotic lesion area in the aorta, which was accompanied by lower hepatic expression of proinflammatory cytokines as well as increased plasma superoxide dismutase activity [94]. These important roles of PPARγ and ApoE as key players within the antiproliferative BMP2/BMPR2-PPARγ-ApoE axis were first demonstrated in HPASMC [28].

## 7. PPARγ in Renal Ischemia Reperfusion Injury

One of the main reasons of acute kidney injury (AKI) is renal ischemia reperfusion injury (IRI), leading to the overproduction of reactive oxygen species (ROS) early during reperfusion. Pioglitazone-pretreated rats subjected to 40 min renal IRI had a minimal decline in renal function and almost normalized fractionated sodium excretion (FENa) and proteinuria, as compared to nontreated IRI rats. This renoprotective effect was accompanied by PPARγ-mediated inhibition of NMDA receptor function [103]. In the most sensitive proximal tubular epithelial cells, ROS triggers apoptosis. PPARγ was shown to reduce ROS generation in kidney epithelial cells after hypoxia in vitro and pioglitazone pretreatment of mice for one week before renal IR reduced AKI. The protective effect of the PPARγ activation was associated with the upregulation of uncoupling protein-1 (UCP1, member of the mitochondrial anion carrier protein family expressed in the mitochondrial inner membrane) in renal epithelia [104]. During renal ischemia/reperfusion, autophagy modulates the extent of kidney injury [105]. Pioglitazone pretreatment of NRK rat kidney cells substantially reduced hypoxia-/reoxygenation-induced apoptosis, via activation of autophagy through the AMPK-mTOR regulatory axis [106].

## 8. The Role of PPARγ in Transplanted Kidneys

Despite the improved immunosuppressive therapies in the past decades leading to a good control of acute rejection and improving short-term graft survivals, chronic rejection of kidney transplants attributed to chronic allograft nephropathy did not improve significantly. Chronic allograft nephropathy (CAN) is mainly caused by excessive inflammation and fibrosis. Biopsies of transplanted kidneys with chronic allograft nephropathy depict increased vascular and tubulointerstitial PAI-1 (plasminogen activator inhibitor-1, a strong profibrotic molecule) expression that is closely associated with fibrosis severity [107]. In a rat model of glomerulosclerosis induced by subtotal nephrectomy, PPARγ activation reduced PAI-1 expression and ameliorated fibrosis, suggesting that PPARγ exerts a protective role in glomerulosclerotic kidneys by downregulating PAI-1 [108]. Interestingly, PPARγ was found to be upregulated in the same kidney areas where PAI-1 was expressed in human biopsies with CAN, and interstitial macrophages were also PPARγ positive in the fibrotic kidneys. This suggests that PPARγ could be induced as counter-acting response to injury in these kidneys [107].

The potential immunosuppressive and antifibrotic effect of PPARγ was also demonstrated in experimental models of allogenic kidney transplantation. Pharmacological activation of PPARγ preserved kidney function of allografts as well as reducing fibrosis, tubular atrophy, and inflammation [109,110]. Furthermore, PPARγ agonist decreased migration and proliferation of both fibroblasts and macrophages [109].

Still, the long-term survival of allografts following renal transplantation highly depends on development of chronic allograft dysfunction. Using the classical Fisher-to-Lewis renal allograft transplantation model, PPARγ activation by rosiglitazone reduced proteinuria by 30% and also decreased interstitial collagen deposition and expression of profibrotic TGFβ. This was accompanied by the reduced expression of renal inflammatory molecules, reduced NF-kB activity, and also attenuated Smad3 phosphorylation [110].

One of the challenges after organ transplantation is the avoidance of immunosuppressive side effects while inhibiting the rejection of grafts. Side effects of immunosuppression can also include deterioration of renal function, so that the use of the potent immunosuppressant Cyclosporin-A (CsA) is sometimes limited due to its known nephrotoxic side effect. Treatment of rats with PPARγ agonist rosiglitazone appear to protect kidneys from CsA toxicity, associated with a reduction of oxidative stress, renal TGFβ expression, and tubular mitochondrial damage [111].

## 9. Resurrection of the PPARγ Agonist Pioglitazone

The TZD class drug rosiglitazone was presumed to increase cardiovascular mortality, but the FDA dropped this assumption in recent years, after evaluation of the RECORD (Rosiglitazone Evaluated for Cardiac Outcomes and Regulation of Glycemia in Diabetes) trial [112].

Pioglitazone improves the systolic and diastolic LV function in rodents and in patients with [113] and without [114] diabetes. Pioglitazone has fewer off-target effects and a better side-effect profile as compared to rosiglitazone. Of note, genetic variation determines PPARγ function and the antidiabetic drug response in vivo [115]. Certain single-nucleotide polymorphisms modify binding of the transcription factor PPARγ to its target genes, influencing the antidiabetic drug response in mice and affecting the individual risk for metabolic disease in humans [115]. Therefore, natural genetic variations modifying the PPARγ function affect the individual disease risk and drug response.

## 10. Summary and Future Directions

Recent studies using PPARγ agonists—and especially pioglitazone—shed light on multiple pathways that can inhibit or even reverse the pathomechanisms at play in PAH and chronic fibroproliferative kidney diseases. These ways of PPARγ actions are either dependent on or independent of the regulation of cell metabolism. In the lungs for instance, PPARγ activation inhibits canonical TGFβ/Smad3 and noncanonical TGFβ/pSTAT3/pFoxO1 pathways in HPASMC, counteracts BMPR2 dysfunction, and induces the antiproliferative PPARγ/apoE axis. PPARγ activation also improves mitochondrial dysfunction and decreases superoxide production. In the kidneys, pioglitazone ameliorates experimental renal fibrosis by repressing TGFβ/pSTAT3 and TGFβ/EGR1 pathways, reducing podocyte injury and apoptosis—partly through restoration of TRPC6—mediated Ca^2+^ influx. The repression of renal TGFβ/Smad signaling by PPARγ activation inhibits interstitial extracellular matrix (ECM) accumulation and epithelial-to-mesenchymal transition (EMT) in both podocytes and tubular epithelium. Additionally, PPARγ activation reduces inflammation and chronic allograft rejection after experimental kidney transplantation. Recent randomized controlled clinical trials show that PPARγ activation with pioglitazone has beneficial effects in cardiovascular patients without significant adverse effects. The experimental and clinical studies suggest that pioglitazone and other, newly developed PPARγ agonists could become a valuable treatment for PAH and kidney fibrosis.

## Abbreviations

ApoE	apolipoprotein-E
BMP2	bone morphogenetic protein 2
BMPR2	bone morphogenetic protein receptor 2
CAN	chronic allograft nephropathy
CKD	chronic kidney disease
CTGF	connective tissue growth factor
ECM	extracellular matrix
EGF	endothelial growth factor
EMT	epithelial-to-mesenchymal transition
FGF1	fibroblast growth factor-1
HPASMC	human pulmonary arterial smooth-muscle cell
LRP1	low-density lipoprotein receptor-related protein 1 (TGFβ receptor 5/ApoE receptor)
IPAH	idiopathic pulmonary arterial hypertension
LV	left ventricle
PAEC	pulmonary endothelial cell
PAH	pulmonary arterial hypertension
PPARγ	peroxisome proliferator-activated receptor gamma
ROS	reactive oxygen species
RV	right ventricle
TGFβ	transforming growth factor-β
UUO	unilateral ureter obstruction
VSMC	vascular smooth-muscle cell
